# Utilization of Inertial Measurement Units for Determining the Sequential Chain of Baseball Strike Posture

**DOI:** 10.3390/s21093280

**Published:** 2021-05-10

**Authors:** Yun-Ju Lee, Po-Chieh Lin, Ling-Ying Chen, Yu-Jung Chen, Jing Nong Liang

**Affiliations:** 1 Department of Industrial Engineering and Engineering Management, National Tsing Hua University, Hsinchu 30013, Taiwan; bogalin@gapp.nthu.edu.tw (P.-C.L.); s107034560@m107.nthu.edu.tw (L.-Y.C.); yjchen617@ie.nthu.edu.tw (Y.-J.C.); 2 Department of Physical Therapy, University of Nevada, Las Vegas, Las Vegas, NV 89154, USA; jingnong.liang@unlv.edu

**Keywords:** inertial measurement units, visual-motor coordination, eye gaze, baseball swing

## Abstract

The purpose of this study was to employ inertial measurement units (IMU) with an eye-tracking device to investigate different swing strategies between two levels of batters. The participants were 20 healthy males aged 20 to 30 years old, with ten professional and ten amateur batters. Eye gaze position, head, shoulder, trunk, and pelvis angular velocity, and ground reaction forces were recorded. The results showed that professional batters rotated segments more rhythmically and efficiently than the amateur group. Firstly, the professional group spent less time in the preparation stages. Secondly, the maximum angular velocity timing of each segment of the professional group was centralized in the swing cycle. Thirdly, the amateur group had significantly earlier gaze timing of the maximum angular velocity than the professional group. Moreover, the maximum angular velocity timing of the gaze was the earliest parameter among the five segments, and significantly earlier (at least 16.32% of cycle time) than the maximum angular velocity of the head, shoulder, trunk, and pelvis within the amateur group. The visual-motor coordination strategies were different between the two groups, which could successfully be determined by wearable instruments of IMU.

## 1. Introduction

With the advancement of wearable technology, inertial measurement units (IMU) have achieved accurate data acquisition, low battery consumption, and are small enough to be blended into daily activities, such as embedded IMU in a smartphone or smartwatch. Research using wearable devices has been applied in the rehabilitation, diagnosis, or sports field for several decades, which analyzed acceleration, angular velocity, ground reaction forces, or electromyographic signals to evaluate treatment outcomes or performance of athletes [1,2].

Baseball striking is considered a challenging task [3] and requires multiple body part movements, which involve the batter’s motor coordination [4,5]. The striking sequence is a kinematic chain starting from the bottom to the top: knee flexion, hip abduction, waist rotation, shoulder rotation, neck rotation, then upper limb swing [6]. A successful striking requires the dynamic balance ability [6], which involves the coordination between the torso rotation and the appropriate body weight shifting [7]. Further, the visual information of the starting hand position is used to define the subsequent kinematic trajectory of upper limb movements, such as reaching [8,9,10]. Moreover, proprioception is essential to real-time feedback on the body position information [8]. Regarding the baseball batter, the horizontal head and eye movements are used to estimate the location and timing of the strike [3]. After receiving the visual information, batters process these messages through the ability of motor coordination.

Visual–motor coordination plays an important role in the swing, including visual tracking, intercept, and moving objects identification [4]. However, a thorough understanding of visual information can enable athletes to perform visual actions effectively and can positively impact the overall performance [11,12]. Hence, vision and proprioception are both critical to arm the configuration and its corresponding movement. Previous studies used a motion capture system to record the rotation of the torso or analyze the ground reaction forces (GRF) during the swing for the purpose of understanding the swing skill, including swing speed of bat and adjustment of swing timing [13]. Most studies focused on the local segment of the body. For example, the visual search strategy of the athlete prior to receiving a pitched ball [14]; the horizontal head and eye movements as baseball batters take pitches and swing at baseball pitches [3]; the investigation of trunk rotation patterns during baseball batting [15]; the effects of the GRFs on the rotation of the body and the joint torques in baseball tee batting [13]; the body weight shift of batter [6]. The research of the best strike timing was based on the rotation angles [16]. The visual search strategy and the horizontal head and eye movements reported that baseball batters took pitches prior to receiving a patched ball [3,14].

Even though trunk rotation [13,15], joint torques [15], and weight shift [6] were also investigated in baseball batting in separate studies, most previous studies in baseball focused on the local segment of the body regardless of the integrated effects of those critical factors in baseball. The relationship between the aforementioned key factors during striking has still not been systematically investigated simultaneously, which might be due to the lack of instrument integration. Additionally, head movement is one of the kinematic chains during body rotation, but head movement during batting was rarely discussed previously, and coaches tended to emphasize the importance of eye movement [17]. Together, it implied a sequential chain of whole-body motions in baseball strike posture. Since the development and convenience of IMU, embedding the devices in baseball suits is possible, and allows tracking and measuring real-time segment motions to evaluate the strike strategy. Therefore, the present study aimed to investigate the strategy and timing of baseball batters from receiving visual information during preparation, coordinating motion, performing the swing, and restoring the balance via IMU and eye-tracking device instrumental integration.

## 2. Materials and Methods

### 2.1. Participants

Ten amateur (age = 23.8 ± 1.4 years, height = 174.3 ± 4.4 cm, mass = 67.1 ± 8.8 kg) and ten professional (age = 21.5 ± 1.4 years, height = 175.5 ± 4.5 cm, mass = 72.5 ± 9.3 kg) right-handed batters participated in the experiment. Amateur batters had no school baseball team experience in the recent ten years. They were not qualified to join a college baseball team and did not patriciate in any regular baseball training. All professional batters had participated in college baseball teams for 1 to 5 years and were current team members of a college baseball team at the time of participation in the study. The protocol was approved by the Ethics Committee of the National Tsing Hua University (REC: 10811HE101), and all participants provided written informed consent before taking part in the experimental process.

### 2.2. Instruments and Procedures

**Figure 1:**
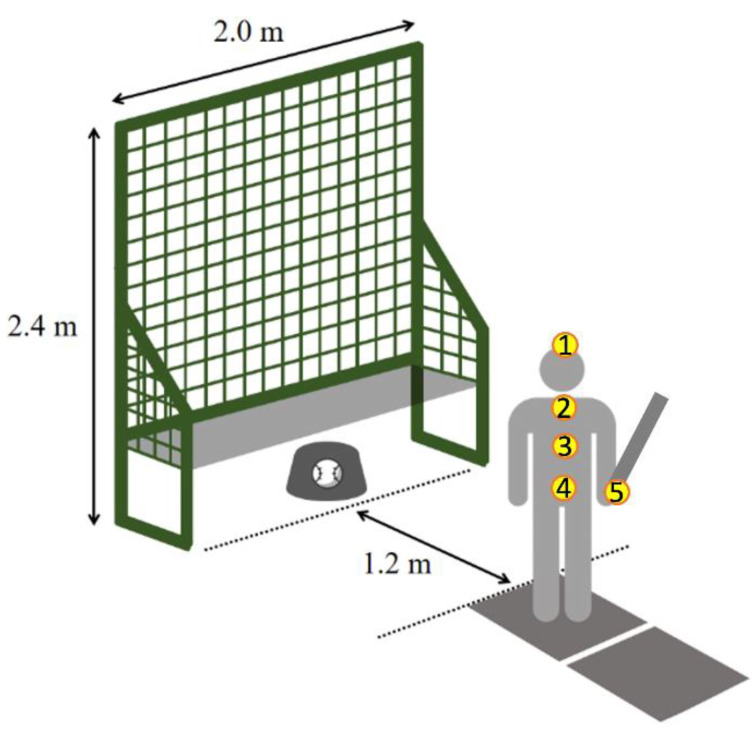
Experimental setting. Five circles represent the placements of five inertial measurement units (IMU). 1—head; 2—vertebra prominens (C7); 3—thoracic vertebrae 10 (T10); 4—sacrum; and 5—the bottom of the bat.

The experimental design is shown in Figure 1. Five IMUs (Delsys Inc., Natick, MA, USA) were placed on the head, vertebra prominens (C7), thoracic vertebrae 10 (T10), sacrum, and the bottom of the bat to measure the rotation of the head, shoulder, and hip, and detect the hitting moment (Figure 1). The head IMU position was set on the intersection point of the center point of two lines. One was the left pre-auricular point (LPA) and the right pre-auricular point (RPA) through the top of the head. The other line was inion to the nasion. An eye tracker (Ergoneers International Holding GmbH, Gewerbering, Germany) was used to detect the fixation point when eyes focused on a target. Six reflective markers were attached to the eye tracker device. Vicon Nexus 2.5 motion capture system (Vicon Industries Inc., Oxford, UK) with eight cameras (Vicon Vero cameras v2.2) calculated the three-dimensional coordinates of the six markers to define the visual axis. Subsequently, the fixation point and the visual axis were used to calculate the vectors as gaze information. Two force plates (Advanced Mechanical Technology Inc., Watertown, MA, USA) embedded in the floor, evaluated shifts in GRFs.

Participants were instructed to stand with one foot on each force plate. A FIELDFORCE baseball throwing machine FTM-240AR was used to toss the ball. The distance between this machine and the participant was 1.2 m. The participants were asked to hit the ball into the net on the throwing machine. The width and height of the net were 2.0 m and 2.4 m, respectively. Each participant warmed up and practiced baseball swings for 15 min, and then completed 30 swings, regardless of whether or not they hit the ball. The batting average was calculated as the percentages of the successful strikes of each participant to compare the strike skill between the two groups.

**Figure 2:**
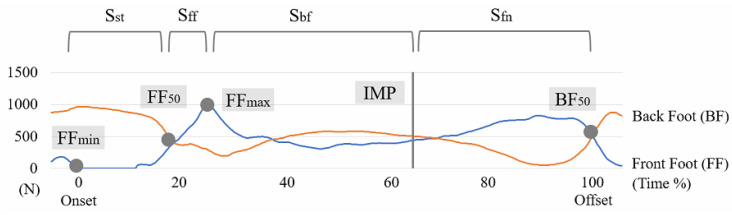
The swing cycle could be divided into four stages by five events. The five events are FF_min_, FF_50_, FF_max_, IMP, and BF_50_ (FF stands for the ground reaction force (GRF) of the front foot, and IMP is the impact moment). The four stages are S_st_ (start stage), S_ff_ (front foot stage), S_bf_ (back foot stage), and S_fn_ (finish stage).

Five event timings of swing were defined in the swing cycle, as shown in Figure 2. First, FF_min_, the minimum GRF of the front foot, or the first frame where it was zero (FF stands for front foot). Second, FF_50_, the GRF of the front foot exceeded 50 percent of the body weight (%BW). Third, FF_max_, the maximum GRF of the front foot. Fourth, IMP (impact), the impact moment of the ball against the bat, which was based on the maximum acceleration data from the IMU sensor. Fifth, BF_50_, the GRF of the back foot exceeded 50% BW or, after IMP, the time closest to 50% BW (BF stands for back foot). These four stages between each event in one swing cycle included S_st_ (S stands for the stage; st stands for a start), S_ff_ (ff stands for front foot), S_bf_ (bf stands for back foot), S_fn_ (fn stands for finish). The swing cycle was defined from FF_min_ to BF_50_. The duration of each stage was displayed as the percentage of the total time.

The difference between the amateur and professional groups focused on three issues, including stage duration, the maximum angular velocity of five segments, and timing of maximum counterclockwise angle of five segments. First, the four stages, including S_St_ duration, S_ff_ duration, S_bf_ duration, and S_fn_ duration, were examined to know whether there were significant differences between the two groups (Figure 2).

Second, the maximum head angular velocity in the five events (FF_min_ to BF_50_) was recorded for comparing the angular velocity difference between the two groups. The other four segments were also investigated, including shoulder, trunk, pelvis, and gaze in the five events (FF_min_ to BF_50_). Third, the timing of the maximum counterclockwise angular velocity of each segment, including head, shoulder, trunk, pelvis, and gaze, were located during the whole swing cycle for comparing the timing of maximum counterclockwise angular velocity between two groups. The timing of maximum counterclockwise angular velocity was displayed as the percentage of the total swing time.

### 2.3. Statistics

An independent sample t-test was used to compare intergroup differences for the percentages of a successful strike and four-time percentage of the swing stage between the amateur and professional groups. The t-test was used to examine the maximum angular velocity of five key event differences between the two groups. Two-factor analysis of variance (ANOVA) was performed to examine the timing of the maximum angular velocity differences between the strike events and skill groups.

## 3. Results

**Figure 3:**
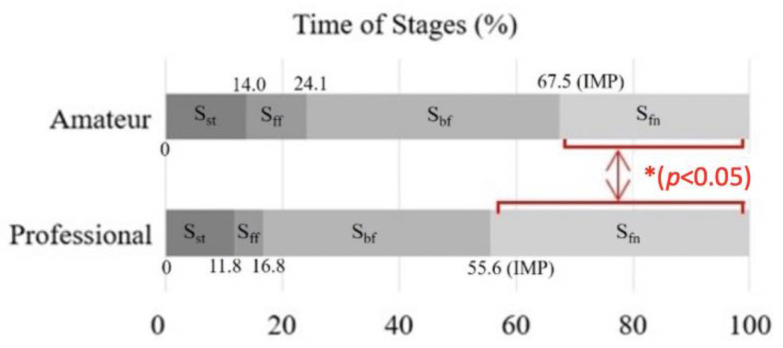
This chart shows the time percentage and timing of the four swing stages of the two groups. In stage S_fn_, the percentage of the amateur batter’s time was significantly lower than professional batters. (*: *p* < 0.05).

The significantly different percentages of the successful strikes confirmed the skill levels between the two groups (t (18) = −2.51, *p* = 0.02, d = 1.24). The percentage of successful strikes by amateur batters (M = 57.5, SD = 17.7) was significantly lower than that of the professional ones (M = 74.9, SD = 12.9). Figure 3 shows the time percentage of the four stages in the two groups. The significant differences between two groups were observed in S_fn_ (t (18) = −3.04, *p* = 0.01, d = 1.36), but not in S_st_ (t (18) = 0.69, *p* = 0.50, d = −0.02), S_ff_ (t (9.32) = 2.07, *p* = 0.07, d = −0.93), S_bf_ (t (18) = 0.95, *p* = 0.35, d = −0.43). In the S_st_ stage, the mean percentage was 13.96 (SD = 8.37) for the amateur batters and 11.82 (SD = 5.21) for the professional batters. In the S_ff_ stage, the mean percentage was 10.13 (SD = 7.74) for the amateur batters and 5.00 (SD = 1.03) for the professional batters. In the S_bf_ stage, the mean percentage was 43.42 (SD = 13.40) for the amateur batters and 38.82 (SD = 7.29) for the professional batters. In the S_fn_ stage, the mean percentage was 32.49 (SD = 10.85) for the amateur batters, which was significantly shorter than for the professional batters (M = 44.36, SD = 5.88).

**Figure 4:**
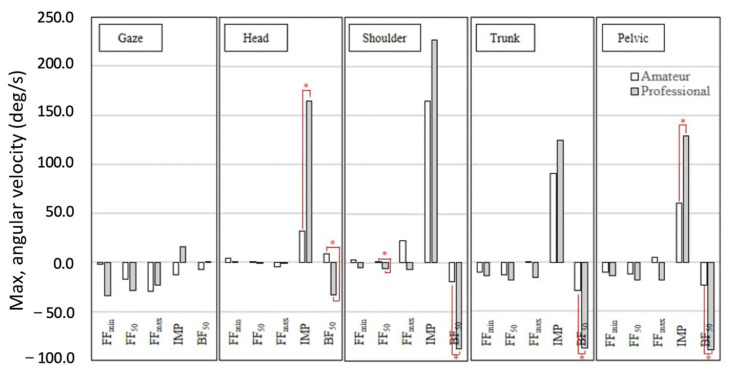
The angular velocity of the amateur and professional groups of the five key events of the head, shoulders, trunk, pelvis, and gaze. (*: *p* < 0.05).

Figure 4 shows the angular velocity of the amateur group and professional group at the five key events, including gaze, head, shoulders, trunk, and pelvis in sequence. In the results of the axial gaze rotation, it was found that the two groups of batters’ gaze angles had no significant differences at FF_min_, FF_50_, FF_max_, IMP, and BF_50_. In the results of the head, it is found that the two groups of batters’ angular velocity had significant differences at IMP and BF_50_, and there was no significant difference at FF_min_, FF_50_, and FF_max_. At IMP, the amateur batters’ angular velocity of the head (M = 31.65, SD = 30.08) was significantly lower than that of professional ones (M = 164.38, SD = 59.99, t (18) = −6.25, *p* = 0.00, d = 2.80); at BF_50_, the amateur batters’ angular velocity of the head (M = 8.56, SD = 22.74) was significantly higher than that of the professional ones (M = −33.05, SD = 15.37, t (18) = 4.79, *p* = 0.00, d = −1.99). In the results of the shoulder, it was found that the two groups of batters’ angular velocity had significant differences at FF_50_ and BF_50_, and there was no significant difference at FF_min_, FF_max_, and IMP. At FF_50_, the amateur batters’ angular velocity of the shoulder (M = 0.73, SD = 5.20) was significantly higher than that of professional ones (M = −6.36, SD = 8.27, t (18) = 2.30, *p* = 0.03, d = −1.03); at BF_50_, the amateur batters’ angular velocity of the shoulder (M = −19.39, SD = 40.87) was significantly higher than that of professional ones (M = −87.77, SD = 47.43, t (18) = 3.45, *p* = 0.00, d = −1.54). In the results of the trunk, it was found that the two groups of batters’ angular velocity had significant differences at BF_50_, and there was no significant difference at FF_min_, FF_50_, FF_max_, and IMP. At BF_50_, the amateur batters’ angular velocity of pelvis (M = −28.55, SD = 29.73) was significantly lower than that of professional ones (M = −87.26, SD = 38.80, t (18) = 3.80, *p* = 0.00, d = −1.70). In the results of the pelvis, it was found that two groups of batters’ angular velocity had significant differences at IMP and BF_50_, and there was no significant difference at FF_min_, FF_50_, and FF_max_. At IMP, the amateur batters’ angular velocity of the pelvis (M = 60.03, SD = 60.68) was significantly lower than that of professional ones (M = 129.14, SD = 68.99, t (18) = −2.38, *p* = 0.03, d = 1.06); at BF_50_, the amateur batters’ angular velocity of the pelvis (M = −22.94, SD = 30.45) was significantly higher than that of professional ones (M = −89.01, SD = 33.76, t (18) = 4.06, *p* = 0.00, d = −2.06).

**Figure 5:**
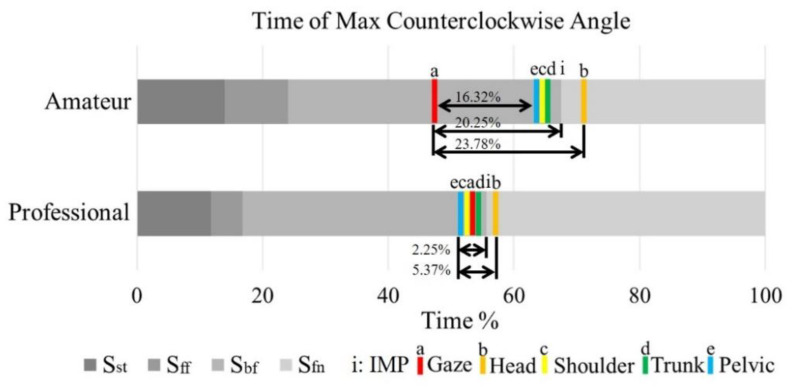
The maximum angular velocity timing of each segment in the swing cycle. The amateur group cost about 23.78% cycle time to complete the maximum angular velocity. However, the professional group cost only about 5.37% cycle time. The maximum gaze angular velocity timing of the amateur group was significantly earlier (16.32% cycle time) than the other segments’ intragroup. Further, the maximum gaze angular velocity timing was earlier, about 20.25% cycle time before IMP of the amateur group. However, only 2.25% cycle time before IMP of the professional group.

Figure 5 shows the maximum angular velocity timing of each segment in the swing cycle, including a: gaze, b: head, c: shoulder, d: trunk, and e: pelvis, which is indicated in the swing stage of the two groups. The amateur group cost about 23.78% cycle time (a to b in Figure 5) to achieve the maximum angular velocity of all segments. However, the professional group cost only about 5.37% cycle time (e to b in Figure 5). The maximum gaze angular velocity timing of the amateur group (at 47.25) was significantly earlier than in other segments (16.32% cycle time). Additionally, the maximum gaze angular velocity timing of the amateur group (at 47.25%) was also earlier, 20.25% cycle time before IMP timing (at 67.50%, see Figure 2).

However, the maximum gaze angular velocity timing (at 53.35%) was only 2.25% cycle time before IMP timing (at 55.60%, see Figure 1) of the professional group. The timing of maximum angular velocity was displayed in Table 1. Two-way ANOVA was performed to examine the skill level and the segmented effect of the maximum angular velocity timing. The result of ANOVA showed that the timing of the maximum angular velocity was significantly different between the two skill level groups (F (1, 18) = 7.05, *p* < 0.001, η_p_^2^ = 0.28). The result of the ANOVA also showed a significant interaction in the timing of the maximum angular velocity between the skill level groups and segments (F (4, 72) = 6.15, *p* < 0.001, η_p_^2^ = 0.26) in Table 2.

**Figure 6:**
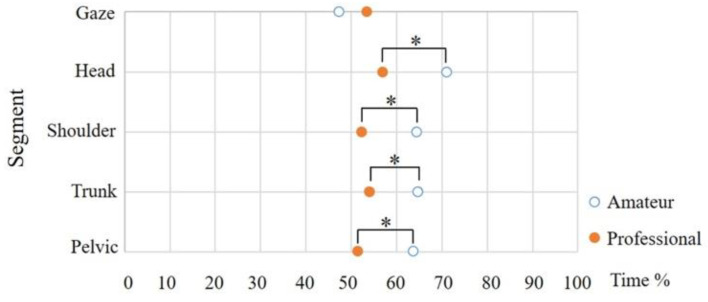
For the amateur group, there was a simple main effect of the segment. The timing of gaze was significantly earlier than the head, shoulder, trunk, and pelvis of the amateur group (*: *p* < 0.001). For the professional group, there was no significant single main effect.

Therefore, simple main effect tests were performed for further examination, and the statistics results are shown in Figure 6. For the gaze, there was no simple main effect of skill level (F (1, 90) = 1.90, *p* = 0.17, η_p_^2^ = 0.02), for the head there was a simple main effect of skill level (F (1, 90) = 10.28, *p* < 0.001, η_p_^2^ = 0.10), the head maximal velocity timing of the amateur group (M = 71.03, SD = 11.49) was significantly later than the professional group (M = 56.84, SD = 4.82). For the shoulder, there was a simple main effect of skill level (F (1, 90) = 7.50, *p* < 0.05, η_p_^2^ = 0.08), the shoulder maximal velocity timing of the amateur group (M = 64.23, SD = 12.11) was significantly later than the professional group (M = 52.11, SD = 5.62). For the trunk, there was a simple main effect of skill level (F (1, 90) = 6.06, *p* < 0.05, η_p_^2^ = 0.06), the trunk maximal velocity timing of the amateur group (M = 64.70, SD = 11.17) was significantly later than the professional group (M = 53.80, SD = 6.22). For the pelvic, there was a simple main effect of skill level (F (1, 90) = 7.47, *p* < 0.05, η_p_^2^ = 0.08), the maximal pelvic velocity timing of the amateur group (M = 63.57, SD = 12.41) was significantly later than the professional group (M = 51.47, SD = 5.69). The following showed the result of the Tukey HSD (Honestly Significant Difference) post hoc. For the amateur group, there was a simple main effect of the segment (F (4, 72) = 13.94, *p* < 0.001, η_p_^2^ = 0.44) in Table 3. The timing of the gaze (M = 47.25, SD = 9.05) was significantly earlier than the head (M = 71.03, SD = 11.49), shoulder (M = 64.23, SD = 12.11), trunk (M = 64.70, SD = 11.17), and pelvis (M = 63.57, SD = 12.41) of the amateur group. For the professional group, there was no significant single main effect (F (4, 72) = 0.77, *p* = 0.55, η_p_^2^ = 0.04).

## 4. Discussion

The successful sequential chain establishment indicated the utilization of IMU for determining if the temporal information could be used to evaluate the strategical swing performance in the baseball strike posture. The batting average (percentage of the successful strike) of the professional group was significantly higher than the amateur group (higher more than 20%), which means the skill level of the strike was really diffident. The swing skill would be affected by the ability of body coordination adjustment [18]. The purpose of this research was to utilize IMU to determine the strategy and timing of baseball batters from receiving visual information during preparation, coordinating the motion, performing the swing, and restoring balance. The IMP timing, the maximum angular velocity at five key events, and the timing of the maximum angular velocity detected from IMU at five key events were discussed in the following.

The timing of the IMP in the professional group was earlier than the amateur group (Figure 3), which indicated that the professional group had less time for hitting preparation than the amateur group. The earlier timing of hitting the ball might be better for a successful strike because the energy transfer in a short time could construct a stronger kinetic chain [15]. The amateur batters had less integrating ability to deal with receiving the batting information, and it might be more difficult to estimate the timing of the swing and hit the ball. Since BF_50_ was defined as GRF exceeding 50%BW after impact, S_fn_ was also related to GRF during the swing process. If batters could correctly shift the weight, they would promote dynamic balance during the swing [6]. The outcomes revealed that professional batters had better performance in dynamic balance, and they took more time to complete the swing after IMP for balance restoring.

The maximum angular velocity of the preparation stages (FF_min_, FF_50_, and FF_max_), IMP stage, and restoring stage (BF_50_) were investigated individually. The professionals demonstrated counterclockwise (negative) angular velocity of gaze, head, shoulder, trunk, and pelvis in the preparation stages. In contrast, the amateurs showed clockwise (positive) angular velocity in FF_min_ of the head and shoulder, in FF_50_ of the head and shoulder, in FF_max_ of the shoulder, trunk, and pelvis. The rotation angular velocity of the shoulder might be an important factor for a successful strike. Motor coordination is the stabilizing ability and mutual agility of various body segments [19]. A previous study indicated a lower torso rotation in the direction of the pitcher (counterclockwise) before the upper torso [20]. The well-trained batters rotated their shoulder counterclockwise before their front foot made contact with the floor [21]. The timing of shoulder rotation in the previous study corresponded with the events of FF_min_ to FF_max_ in our study. Hence, correct shoulder rotation could be considered an important factor for a successful strike. The other possible reason for the FF_50_ difference of the shoulder between the two groups might be temporal awareness, which is the ability to organize the events rhythmically [4]. In addition, the professional batters might have better temporal awareness to predict the baseball location after ball throwing and then perform the larger counterclockwise shoulder rotation for the strike than amateur batters in the preparation stages.

The difference between the extreme angular velocity timing of amateur batters’ heads and other body segments also showed lack of synchronization in coordination. The professional group could produce larger and earlier angular momentum and kinematic energy transition than the amateur group [15,17], particularly in the IMP stage and the restoring stage (BF_50_). Amateur batters first rotated the gaze to maximum angular velocity during the entire swing. In contrast, the professional batters rotated the gaze to the maximum angular velocity after pelvis and shoulders. The timing difference between the two groups might be due to their coordination ability. The professional batters focused on overall movement and perception when striking; in contrast, the amateur batters focused on each step of the strike [22]. In addition, striking involved batters’ innate eye-hand and eye-foot coordination [4]. The reflection of visual stimuli affected the reaction time of segment movements [23]. Professional batters had better visual–motor coordination than amateur batters, which were used to track the ball and perceived information more clearly. Thus, it was easier for professional batters to link the estimated location of the ball with their responding motion consistently and adjusted the extreme rotation time point of each body segment in a short period, consequently demonstrating better performance.

The study had some limitations. First, only ten professional and ten amateur players participated in the experiment, which might not represent the performance of the population. Second, since the difficulties of having participants and different successful strikes, there were insufficient data to conduct the machine learning approach for classification evaluations. However, several outcomes showed statistically significant differences between the two groups, which might somehow reveal their differences and represent their characteristics at certain levels.

## 5. Conclusions

The successful sequential chain was established by the characteristics of IMU and showed different strategic behaviors of baseball strike posture. The professional batters spent a shorter time before IMP and rotated segments counterclockwise, especially in the shoulder, producing larger angular momentum and efficiently transmitting kinetic energy. Furthermore, the timing of the maximum angular velocity of each segment was centralized in 5.37% of the total cycle time of the professional group; however, the amateur group cost 23.78% of the total time. Moreover, only the maximum angular velocity timing of the gaze in the amateur group was significantly earlier than the professional group. The professional batters demonstrated better motor coordination and temporal awareness than amateur batters. An important direction of future work would consider these labeled data of the professional and amateur performances as dataset creation. Finally, the utilization of IMU could be real-time for evaluating the ability of visual-motor coordination for future strike practice, even in playing directly. This online data collection with the dataset creation from the present study could further conduct machine learning approaches for skill-level classification.

## Figures and Tables

**Table 1:** The mean (and SD) maximum angular velocity timing of each segment.

Skill Level	*n*	Gaze	Head	Shoulder	Trunk	Pelvis
		M (SD)	M (SD)	M (SD)	M (SD)	M (SD)
Amateur	10	47.25 (9.05)	71.03 (11.49)	64.23 (12.11)	64.7 (11.17)	63.57 (12.41)
Professional	10	53.35 (14.64)	56.84 (4.82)	52.11 (5.62)	53.8 (6.22)	51.47 (5.69)

**Table 2:** Two-way ANOVA evaluations of group and segment effects.

Source of Variation	SS	df	MS	F	*p*	η_p_^2^
A Skill level	1866.33	1	1866.33	7.05	<0.001	0.28
Error	4764.13	18	264.67			
B Segment	1923.48	4	480.87	8.54	<0.001	0.32
A*B	1385.25	4	346.31	6.15	<0.001	0.26
Error	4052.28	5472	56.28			

**Table 3:** Simple main effects on the group and segments.

Source of Variation	SS	df	MS	F	*p*	η_p_^2^
Skill level (A)						
Gaze (B1)	186.05	1	186.05	1.90	0.17	0.02
Head (B2)	1006.78	1	1006.78	10.28	<0.001	0.01
Shoulder (B3)	734.47	1	734.47	7.50	<0.05	0.8
Trunk (B4)	594.05	1	594.05	6.06	<0.05	0.06
Pelvic (B5)	732.05	1	732.05	7.47	<0.05	0.08
Error	8816.22	90	97.96			
Segment (B)						
Amateur (A1)	3137.10	4	784.27	13.94	<0.001	0.44
Professional (A2)	173.20	4	43.30	0.77	0.55	0.04
Error	4052.16	72	56.28			

## Data Availability

Data available on request due to restrictions of privacy and ethical issue.
